# Auxin–cytokinin interactions in the regulation of correlative inhibition in two-branched pea seedlings

**DOI:** 10.1093/jxb/ery117

**Published:** 2018-03-24

**Authors:** Andrey A Kotov, Liudmila M Kotova

**Affiliations:** Institute of Plant Physiology, Russian Academy of Sciences, Botanicheskaya, Moscow, Russia

**Keywords:** Correlative inhibition, cytokinins, indole-3-acetic acid, *Pisum sativum*, transport, two-branched pea seedlings, xylem sap

## Abstract

A model system of 10–12 day-old, two-branched (2-B) pea (*Pisum sati*v*um* L. cv. Adagumsky) seedlings was used to study the roles of endogenous auxin indole-3-acetic acid (IAA) and cytokinins (CKs) in the interaction between the shoots. The IAA export activity (IEA) from shoots was 2-fold higher in one-branched (1-B) plants with one shoot removed than in the 2-B plants, while tZ-type cytokinin contents in xylem sap were 4-fold greater in the 1-B plants than in 2-B plants. Exogenous 6-benzylaminopurine introduced into the vascular stream of one shoot enhanced its IEA. Therefore, xylem cytokinin appears to control both growth and IEA in branches. In the hypocotyls of 1-B and 2-B plants, IAA contents were equal in both cases, while the levels of tZ-type cytokinins were different. These data do not agree with the well-supported role of auxin in down-regulating CK content. The observed paradox may be explained by assuming that a steady-state IAA level in the hypocotyls is feedback regulated via xylem cytokinin, which controls the delivery of IAA from the shoots. As a result, the level of IAA in the hypocotyl is most likely maintained at a threshold below which a decrease in auxin content can switch on CK synthesis that will increase xylem cytokinin levels, thereby stabilizing the level of IAA in the hypocotyl. Therefore, our results suggest that correlative inhibition in the 2-B pea system is a function of an IAA/CK feedback loop, in which cytokinin essentially acts as a second messenger for IAA.

## Teaser

In a two-branched pea system, growth and IAA export from branches are controlled by a xylem cytokinin feedback loop regulated by apical auxin.

## Introduction

Plants can be considered to be ‘competing populations of redundant organs’ (Sachs, 2002). Early experiments on a model system of two-branched (2-B) pea seedlings, in which one shoot was correlatively inhibited by the other, revealed a positive correlation between shoot growth and export from the shoot of the auxin indole-3-acetic acid (IAA) (Morris, 1977; Morris and Johnson, 1990; Li and Bangerth, 1999). Removal of the dominant shoot leads to increased IAA export from the repressed shoot; this observation led to the conclusion that active auxin transport from shoots is both an essential attribute of shoot growth and an important means to affect the growth of other shoots. Accordingly, it was found that the correlatively inhibited branch in 2-B pea seedlings had active IAA efflux carriers but was unable to directionally transport IAA, while both auxin transport and branch growth could be restored by removal of the dominant branch (Morris, 1977; Morris and Johnson, 1990). By analogy, following decapitation in pea, the axillary buds are released from auxin-mediated apical dominance, which is correlated with the restoration of directional auxin export by subcellular polarization of PIN efflux carriers (Kalousek *et al.*, 2010; Balla *et al.*, 2011; Balla *et al.*, 2016). To explain how auxin transport can be involved in correlative inhibition (CI) among the shoots, two possible models were proposed.

According to the auxin transport model, CI between shoots (e.g. two branches) results from competition between their auxin sources (shoot apices) when auxin transport from a dominant shoot can somehow inhibit auxin transport from subordinate shoot(s) without the involvement of any second messenger. Initially, it was suggested that such auxin transport competition occurs by some kind of auxin transport autoinhibition (ATA) at the junction of two branches. (Li and Bangerth, 1999; Bangerth *et al.*, 2000). Applying the competitive principle in auxin canalization previously presented by Sachs (1969), Bennett *et al.* (2006) proposed that the earlier-occurring IAA export from the dominant organ inhibits later IAA export from subordinate organs due to the saturation of auxin transporters in the polar auxin transport stream of the main stem. However, auxin transport at physiological concentrations was not found to be limited by the availability of auxin carriers (Brewer *et al.*, 2009; Renton *et al.*, 2012). Later, Prusinkiewicz *et al.* (2009) showed that the assumption of saturation, while intuitive, is not required, and presented a computation model in which competition can emerge from positive feedback between auxin flux and polarization of active auxin transport according to Mitchison’s equations (Kramer, 2009). This model successfully explained CI specificity in various branched Arabidopsis mutants on the basis of specificity in their PIN-dependent IAA transport, and accounted for ‘the apparent paradox that increased branching can be achieved either by decreasing accumulation of active PINs on the membrane, as in *tir3*, or by increasing accumulation, as in the *max* mutants’ (Prusinkiewicz *et al.*, 2009). Despite the above arguments in favor of an auxin transport hypothesis, at the molecular level, flux-based principles of auxin efflux carrier polarization have still not been elucidated (Kramer, 2009; Tanaka *et al.*, 2013), and it is not known how auxin in the polar auxin transport stream is able to inhibit auxin export from branches (Bennett *et al.*, 2014).

The second messenger model proposed that basipetal IAA transport from one branch can inhibit IAA export from the other, acting through cytokinins (CKs). CK synthesis or xylem content can be regulated by negative feedback from IAA exported from the shoots (Bangerth, 1994; Li *et al.*, 1995; Kotova *et al.*, 2004; Nordstrom *et al.*, 2004), and in this case CKs function as intermediary signals by regulating auxin export from the branches (Li and Bangerth, 1992; 2003). Consistent with this model, the expression of two CK biosynthesis genes, *Ps*IPT1 and *Ps*IPT2, in pea internodes was shown to be activated by decapitation and suppressed by apical application of exogenous auxin (Tanaka *et al.*, 2006; Dun *et al.*, 2012). The precise mechanism of an auxin-dependent repression of IPT genes remains unclear, and auxin-responsive factors such as SHY2/IAA3 and Aux/IAA9 (Wang *et al.*, 2005; Weijers *et al.*, 2005; Chapman and Estelle, 2009; Sakakibara, 2010) may be involved in this process. In turn, the synthesis of *trans*-zeatin (tZ)-type CKs is most likely an auxin-regulated process. This follows from the fact that in Arabidopsis seedlings the expression of the tZ-type CK biosynthesis gene *CYP735A* significantly decreased 1 h after treatment with auxin (Takei *et al.*, 2004). Li and Bangerth (1992; 2003) showed that CKs can activate auxin export from the shoot apex. In Arabidopsis, CK appears to promote the expression of *IAA17/AXR3* and *SHY2*, but the roles of these signaling genes, especially *SHY2*, in auxin biosynthesis is not clear (Jones *et al.*, 2010). On the other hand, it has been reported that treatment of axillary buds in intact pea plants with a synthetic CK, 6-benzylaminopurine (BA), increases the expression of genes encoding auxin influx (*Ps*AUX1) and efflux (*Ps*PIN1) carriers, along with polarization of *Ps*PIN1 protein in the buds (Kalousek *et al.*, 2010). Interestingly, direct application of IAA to these buds led to higher *Ps*AUX1 and *Ps*PIN1 expression, but did not cause the repolarization of *Ps*PIN1 proteins (Kalousek *et al.*, 2010), thus suggesting a specific role for CK in polar auxin transport regulation.

Many authors have argued that both models are consistent with the concept of a hybrid model of branching in which both second messenger and auxin transport effects occur (Dun *et al.*, 2013; Rameau *et al.*, 2015; Seale *et al.*, 2017). Results obtained in pea seedlings with partially reduced shoot tips indicated that the growth rates of axillary buds are closely correlated with the CK/IAA ratio in internode tissues (Kotov and Kotova, 2000), data that might support the hybrid model of apical dominance. In the present study, using 2-B pea seedlings as a model system, we provide evidence that CI between shoots is a direct consequence of the interactions between IAA and CK and, hence, xylem CK appears to control both growth and IAA export from branches.

## Materials and methods

### Production of two-branched, one-branched, and branchless pea plants

Seeds of *Pisum sati*v*um* L. cv. Adagumsky were soaked and germinated between two layers of filter paper moistened with distilled water for 3 days at 25 °С in the dark (Kotov and Kotova, 2015). After 4 days, the epicotyl was removed from the cotyledonary node to induce the growth of two axillary buds at this node. Forty seedlings were grown in tap water in a 0.5 l vessel at 20 ± 1°С under fluorescent light providing an intensity of 135 μmol m^–2^ s^–1^, with a 14 h day/10 h night photoperiod. Four days later, the seedlings with two equal cotyledonary shoots (2-B) were selected and transferred to 0.4 l trays (6 × 16 × 6 cm) with tap water, with nine plants per tray, under the same conditions. To obtain one-branched (1-B) or branchless (0-B) plants, one or two shoots were removed from 8-, 10-, or 14-day-old 2-B plants. Two days later, in the 10-, 12-n or 16-day-old plants, samples of hypocotyl tissues, xylem sap, and shoot diffusates were taken depending on the experiment.

### Shoot growth measurements

For shoot growth analysis, shoot lengths of 2-B and 1-B plants produced on day 8 were measured daily during days 8–11 using a horizontal calibrated stereomicroscope. The shoot relative growth rate (RGR_(*i*)_) at each day *i* was calculated as RGR_(*i*)_=[(L_(*i+1*)_*–* L_(*i*)_)/L_(*i*)_]/24 h, where L_(*i*)_ and L_(*i+1*)_ were shoot lengths on day *i* and 24 h later, respectively. The mean RGR value and standard error were determined from nine biological replicates for each treatment.

### Hormonal treatments

For vascular supply, in 10-day-old 2-B plants a thread submerged in 1.5 ml of 1 µM or 10 µM BA (Serva, Heidelberg, Germany) or control solutions (all containing 0.1 % DMSO) was extended with a needle through the stem base of one cotyledonary shoot (Fig. 3) according to Gomez-Roldan *et al.* (2008).

### Estimation of IAA export activity in the cotyledonary shoots

**Fig. 1.**
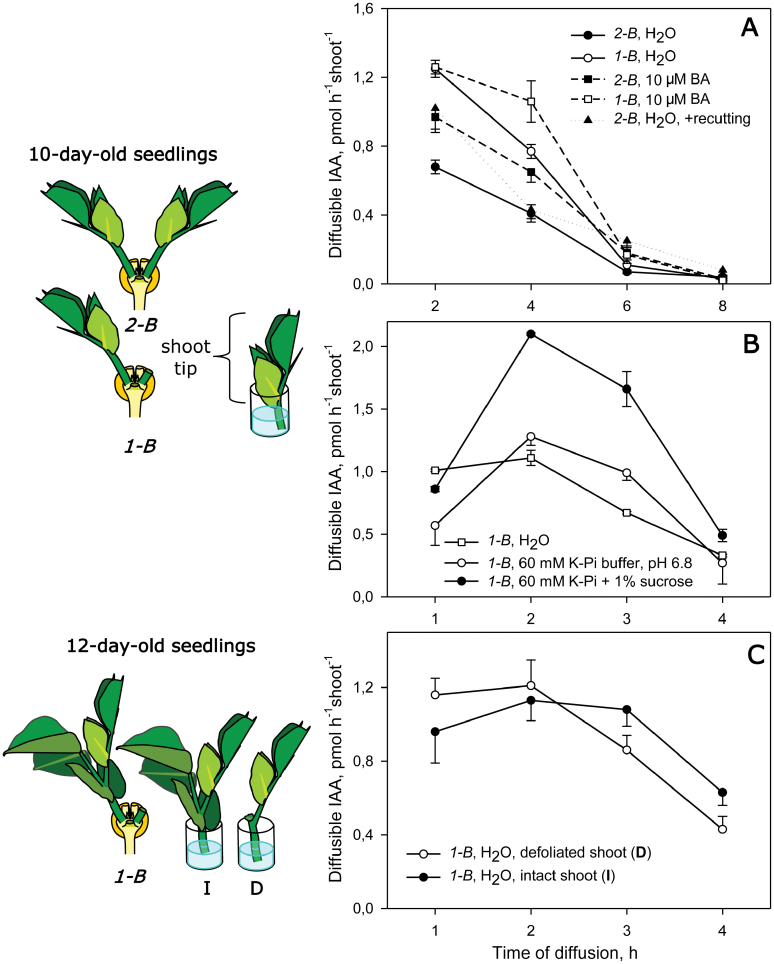
(A, B) Influence of the medium on the time course of IAA diffusion out of excised shoot tips (diffusible IAA) from 10-day-old two-branched (2-B) and one-branched (1-B) pea seedlings. (C) Effect of defoliation on IAA export from shoots of 12-day-old 1-B plans. Schematic views of the experimental set-up for IAA export measurements is shown on the left. The 2-B plants were prepared by removing the epicotyl from 4-day-old seedlings to induce the growth of two axillary buds at the cotyledonary node. 1-B plants were then produced by removing one branch from 2-B plants at day 8 or 10. After 2 days, diffusible IAA from excised shoot tips of 10- or 12-day-old 2-B and 1-B plants was collected into 120–150 µl of aqueous medium stepwise during an incubation period of 2 h (A) or four 1 h periods (B, C), and IAA was measured by ELISA. Data are expressed as the mean ±SE of three samples pooled from six shoot diffusates. BA, 6-benzylaminopurine; K-Pi, potassium phosphate buffer.

Export of IAA from shoot apices (diffusible IAA) was measured by a diffusate method. Unless otherwise stated, each shoot tip (shoot apical bud with uppermost leaf) (Fig. 1A, B) was cut off and its basal end was placed individually into 120–150 µl of distilled water. The samples were incubated in a humid box for 2 h at 20 ± 1 °С in darkness, and then IAA-containing diffusates from each separate shoot tip were stored at –20 °C before analysis. The shoot tips were cut off from the underlying internode and weighed. The diffusates from 5–6 shoot tips of similar weight (not exceeding ±5 mg) were pooled and subjected to ELISA for measuring diffusible IAA. The correlation of IAA amount with average shoot tip weight was calculated by linear regression analysis (SigmaPlot 11 for Windows 7) conducted on the 5–10 data points for each treatment. The shoot IEA was expressed as the amount of diffusible IAA in the shoot tip having a standard weight of 45 mg, and calculated using the linear regression equations in SigmaPlot 11 with standard error of the estimate.

**Fig. 2.**
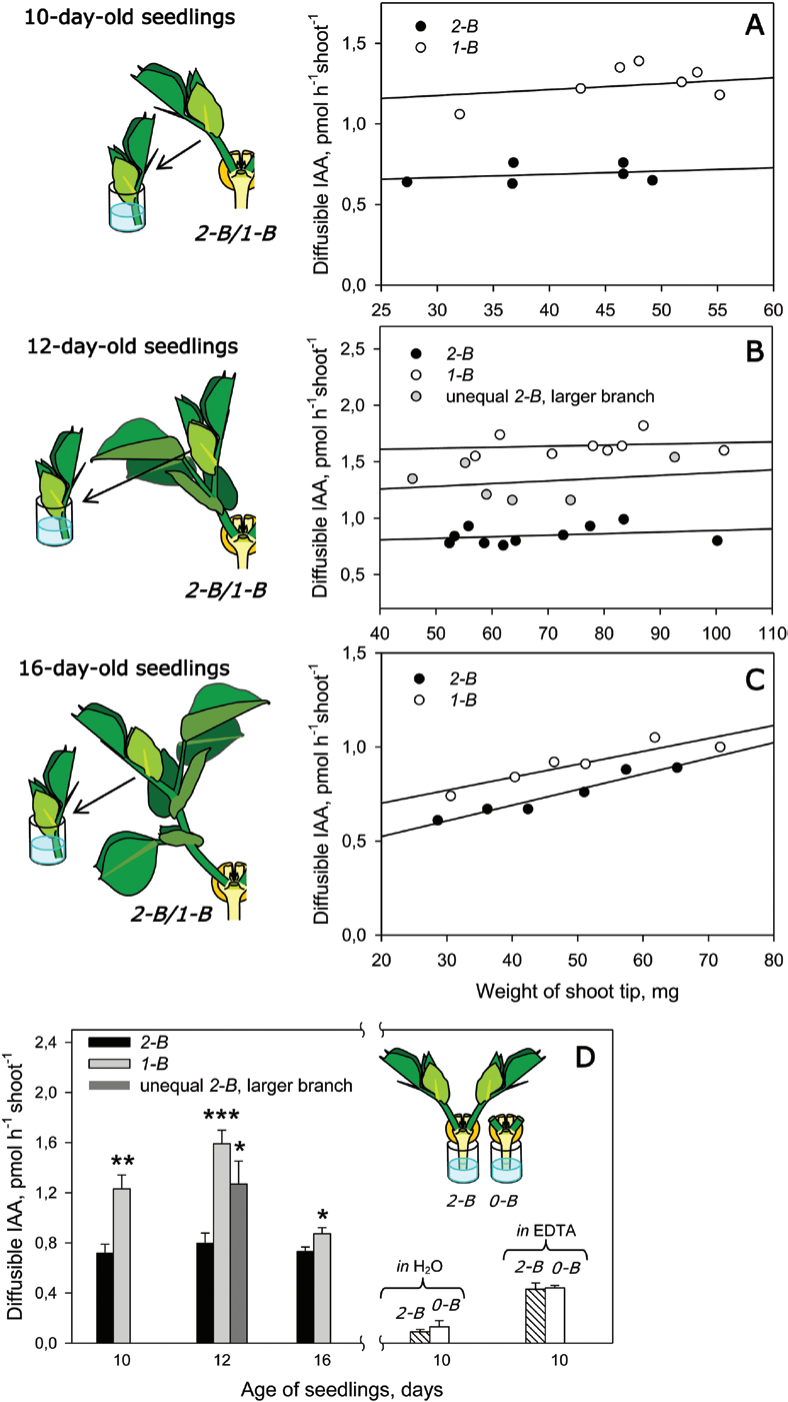
IAA export from excised shoot tips as a function of the shoot tip weight for (A) 10-day-old, (B) 12-day-old, and (C) 16-day-old 2-B and 1-B pea seedlings. A schematic of the experimental set-up is shown on the left. (D, left side) Age dependence of IAA export activity from 2-B and 1-B shoot tips with a standard weight of 45 mg. (D, right side) IAA transport from intact 2-B and 0-B de-rooted seedlings collected for 2 h into distilled water or 0.5 mM EDTA, pH 7.05 (150 µl per plant). Plants were prepared as in Fig. 1 and diffusible IAA from each excised shoot tip was collected into 120–150 µl of distilled water for 2 h. Diffusates from six shoot tips with similar weight (≤5 mg) were combined for ELISA. In (A–C), the data were analyzed using linear regression (SigmaPlot 11 for Windows 7); in (D, left side), values were interpolated by linear regression of existing data points in (A–C) ±SE. In (D, right side), data are expressed as the mean ±SE of three samples pooled from five shoot diffusates. In (D), asterisks indicate statistically significant differences compared with 2-B plants: *P<0.05; **P<0.01, ***P<0.001.

In some experiments, 2 h diffusates were collected from whole 10-day-old, 2-B and 0-B seedlings that were previously de-rooted 1.5 cm below the cotyledonary node (Fig. 2D, right panel) by immersing the basal end in 150 µl of distilled water or in 0.5 mM EDTA (pH 7.05) to measure possible phloem IAA transport (Kotov and Kotova, 2000; Jager *et al.*, 2007).

### Collection of xylem sap and estimation of acropetal CK transport

Xylem sap was collected in 10-day-old 2-B, 1-B, and 0-B plants by a vacuum-suction technique (Kotov and Kotova, 2015). In short, root plus hypocotyl or root alone was cut off immediately below or 1.5 cm below the cotyledonary node, respectively, and a flexible silicone tube (length 17 mm, internal diameter 2–2.5 mm) was stretched over the stump of the hypocotyl or root. The other end of the tube was attached to a xylem sap receiver (1 ml pipette tip) connected to an air duct system in which negative pressure was generated by a water-suction pump controlled by a manometer and manually adjusted using an inlet valve. Suction was applied for 30 min at a pressure of –0.6 to –0.8 MPa. Xylem sap samples collected from each plant were transferred into pre-weighed 0.5 ml eppendorf vials, weighed, and stored at –20°С.

For CK analysis, xylem sap collected from the 2–4 plants exuding similar amounts of sap were pooled, diluted with distilled water (1:2), and subjected to ELISA with anti-zeatin riboside (ZR) or anti-isopentenyladenosine (iPR) antibodies. Results were expressed in ZR or iPR equivalents. For each treatment, the amount of tZ-type CK measured in 12–18 samples was correlated with the corresponding sap flow rate using linear regression (SigmaPlot 11 for Windows 7). As transpiration rates (<0.01 µl s^–1^) are very low in 10-day-old pea plants (Kotov and Kotova, 2015), the real concentration of tZ-type CK in xylem sap *in vivo* (the acropetal transport of CK) could be approximated as the concentration of CK in xylem sap at near-zero levels of sap flow rate. The values presented in the summary diagram of the xylem CK levels in Fig. 4C were extrapolated to null sap flow rate by linear regression and calculated with standard error of the estimate.

For column chromatography of tZ-type CK, xylem sap was buffered with phosphate-buffered saline (PBS; 0.01 M sodium phosphate buffer with 0.15 M NaCl, pH 7.4). A sample aliquot of 500 μl was applied to a 1.5 ml Toyopearl HW 40F column (Toso, Japan), which was then eluted with PBS (Kotov and Kotova, 2000; 2015), and 0.5 ml fractions were analyzed by ELISA with anti-ZR antibodies.

### Collection and preparation of hypocotyl samples for IAA and CK analyses

Hypocotyl segments (2–3 mm long) from 10-day-old 2-B, 1-B, or 0-B pea seedlings were cut 2–3 mm below the cotyledons, frozen, and stored in liquid nitrogen. Using four replicates per treatment (10 segments per replicate, one segment per plant), samples were extracted and purified for ELISA (Kotov and Kotova, 2000). Briefly, each frozen tissue sample (150–250 mg) was homogenized and extracted overnight at 4 °С with 5 ml of 80% (v/v) methanol containing 200 mg l^–1^ butylated hydroxytoluene and 100 mg l^–1^ ascorbic acid, with shaking. The extract was filtered and passed through a 50 mg Porolas-TM cartridge (hydrophobic sorbent with an average pore size of 4.24 nm, 50–250 μm particle size, OmskKhimProm, Russia). The filter and cartridge were washed with 0.5 ml or 1 ml of 80 % methanol, respectively, and the washings were combined with the corresponding eluents, giving a final volume of 6.5 ml per sample. These extracts were evaporated down to the aqueous residue (~0.5 ml per sample) at 40 °C under a stream of nitrogen. The residue was diluted to a final volume of 2ml of 50 mM potassium phosphate buffer (pH 3.0) and this aqueous sample was purified on a column (internal diameter 8 mm) containing 1 ml of pre-swollen polyvinylpolypyrrolidone (PVPP, Sigma, USA) to remove polyphenolic compounds. The PVPP column was washed with 6 ml of the same buffer. The washes were combined with the column eluent to obtain the total CK fraction (8 ml). After washing the PVPP column with 1 ml H_2_O and 0.5 ml PBS (pH 7.4), IAA was eluted with 3 ml PBS and the obtained IAA fractions were subjected to ELISA with anti-IAA antibodies.

A 1 ml aliquot of the total CK fraction was neutralized to pH 7.4 with 200 μl of 200 mM K_3_PO_4_, and subjected to ELISA with anti-iPR antibodies. The remaining 7 ml was used to isolate the fraction containing both zeatin and ZR (Z/ZR) from the total CKs on a C18 column (300 mg sorbent was combined from three Amprep С18 100 mg columns (# RPR 1900, Amersham, UK) into one column). For this, the sample was loaded on to the C18 column, and after column washing with 2 ml 50 mM potassium phosphate buffer (pH 3.0) and 2 ml H_2_O, Z/ZR was eluted with 5.5 ml 0.2 N acetic acid in 20% methanol into a Porolas-TM cartridge (0.8 ml). From this cartridge Z/ZR was collected with 4 ml methanol, evaporated to dryness at 40 °C under a stream of nitrogen, redissolved in 1 ml PBS, and stored at –20°С prior to ELISA. Aliquots of the obtained Z/ZR fractions were chromatographed on a 1.5 ml Toyopearl HW 40F column with PBS (pH 7.4) as an eluent (as described above). The recoveries of IAA, ZR, and iPR standards after the purification procedure were 88%, 85%, and 84%, respectively. No compensation was made for their losses in our experiments.

### ELISA of IAA, Z-type, and isopentenyladenine-type cytokinins

Antiserum against Z/ZR, iP/iPR, and IAA was produced by immunizing rabbits with bovine serum albumin conjugated with ZR, iPA, or IAA, respectively (Kotov and Kotova, 2000). Antigens for coating plates used ovalbumin as the carrier protein, and dihydrozeatin riboside was conjugated with ovalbumin for heterologous assay of Z/ZR (Kotov and Kotova, 2015). Competitive indirect ELISA was performed on 96-well microtiter plates (Costar # 9018, USA) (Kotov and Kotova, 2000; 2015). For the detection of primary antibodies, horseradish peroxidase-conjugated sheep anti-rabbit IgG was used (Medgamal, Moscow, Russia). Non-specific interference was tested by adding known amounts of a hormone standard into the sample (Neuman and Smit, 1990). The added/found ratio was within 100 ± 15% for all types of sample (diffusates, xylem sap, and hypocotyl tissue).

### Statistical analysis

**Fig. 3.**
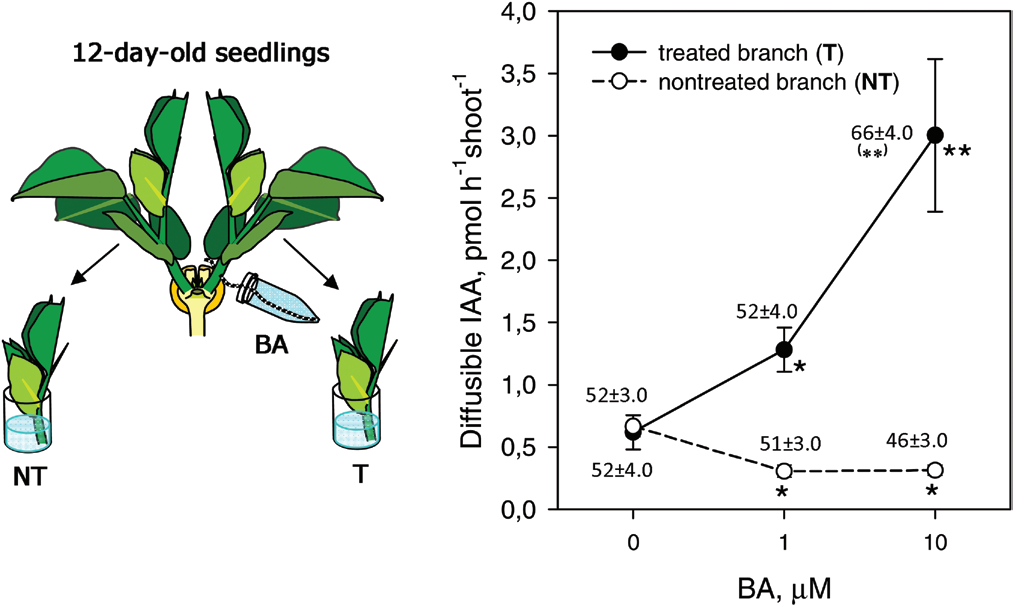
Effects of 6-benzylaminopurine (BA) treatment on the IAA export activity of shoots of 12-day-old, 2-B pea seedlings. BA was supplied into the vascular stream of one shoot (treated shoot; T). NT, non-treated shoot. A schematic of the experimental set-up is shown on the left. 2-B plants were prepared as for Fig. 1. For vascular supply, a thread submerged in 1.5 ml of 0, 1, or 10 µM BA solution (all containing 0.1% DMSO) was passed with a needle through the stem of one shoot at its base. Two days later, the IEA of T and NT shoots was determined by collecting 2 h diffusates from excised shoot tips into distilled water and subjecting the diffusates to ELISA. For each treatment, the amount of IAA measured in 5–6 diffusate samples pooled from five shoot tips was correlated with the average weight of corresponding shoot tips using linear regression (SigmaPlot 11 for Windows 7). IEA values estimated as diffusible IAA in the shoot tips with a standard weight of 45 mg were calculated using the linear regression equations (±SE). Asterisks indicate statistically significant differences compared with control (0 µM BA): *P<0.05, **P<0.01. The fresh weights of shoot apical buds were measured at 12 days (2 days after treatments) and are shown above the points as mg per bud ±SE (n=30); **P<0.01.

**Fig. 4.**
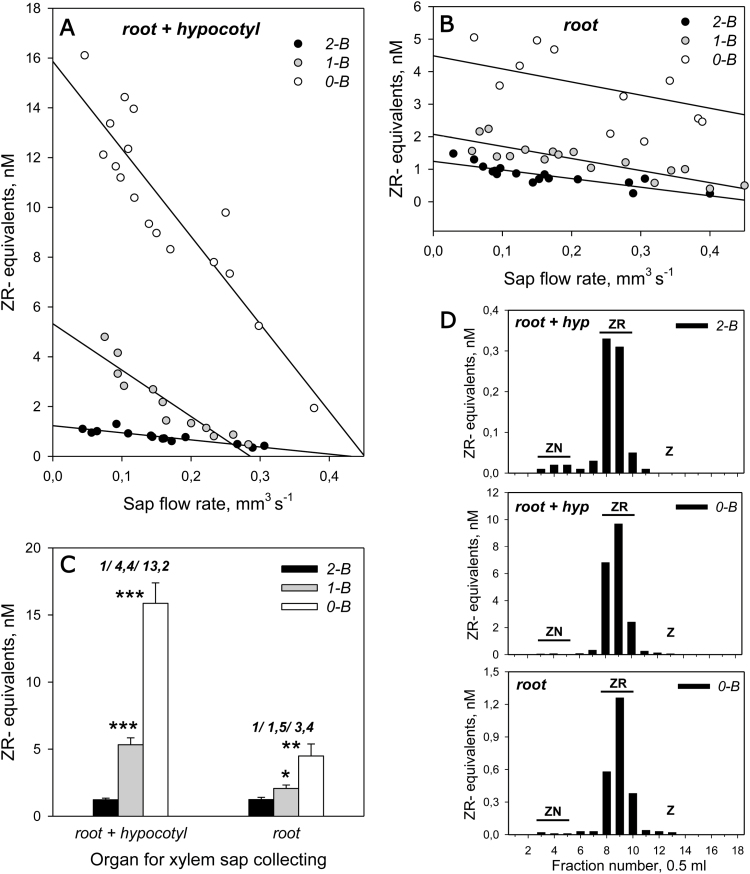
Concentrations of tZ-type CKs in the xylem sap from root plus hypocotyl (A) or from root alone (B) as a function of sap flow rate in 10-day-old 2-B, 1-B, and 0-B pea seedlings. (C) Summary diagram of xylem CK levels extrapolated to null sap-flow rate from data in (A) and (B) with the ratios of 2-B/1-B/0-B values shown above the bars. (D) Immunohistogram of the reactivity of anti-ZR antibodies against the Toyopearl HW 40F column-separated fractions of xylem sap. Plants were prepared as in Fig. 1 at day 8, and two days later xylem sap samples were taken by cutting off the roots, with hypocotyl (A) or without (B). Xylem sap was collected for 30 min under reduced pressure. Sap samples from 2–4 plants displaying similar sap flow rates were pooled and subjected to ELISA. Values are expressed in ZR equivalents. For each treatment (A, B), the relationship was fitted with linear regression giving *P*-values of ≥0.998 at α=0.05 for all variants, except for 0-B plants in (B), where *P*=0.739. The values in (C) extrapolated to null sap-flow rate are closely related to xylem CK levels *in vivo* given near-zero transpiration rates (<0.01 mm^3^ s^–1^) previously observed in 10-day-old pea seedlings by Kotov and Kotova (2015). Asterisks indicate statistically significant differences compared with 2-B plants: *P<0.05, **P<0.01, ***P<0.001. In (D), the column chromatography of xylem sap was performed using PBS (pH 7.4) as eluent buffer. Fractions were analyzed by ELISA; the elution of CK standards is indicated by horizontal bars: Z, *t*-zeatin; ZN, zeatin nucleotide; ZR, zeatin riboside.

**Fig. 5.**
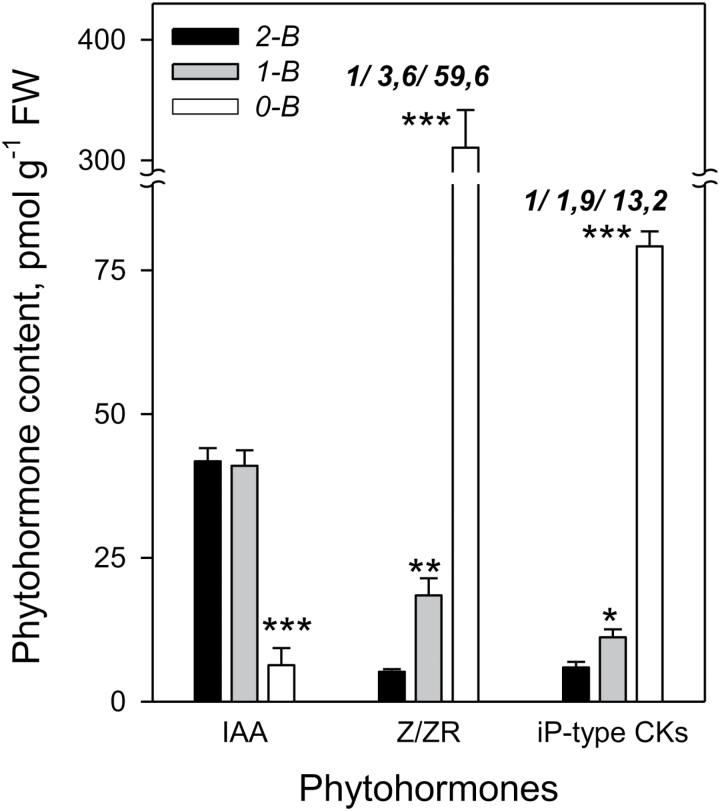
IAA and CK contents in the hypocotyl tissues of 10-day-old 2-B, 1-B, and 0-B pea seedlings. The ratio of 2-B/1-B/0-B plant CK levels is shown above the bars. 2-B, 1-B, and 0-B plants were prepared as for Fig. 4, and hypocotyl samples from all treatments of 10-day-old plants were collected for analysis. IAA and tZ+iP-type CKs were purified and fractionated on PVPP columns, whereas Z/ZR were separated from the total CK fractions on Amprep С18 columns. IAA and CKs were measured by ELISA and expressed as ZR or iPR equivalents per g fresh weight. Data are the means of four replicates (10 plants in each) ±SE. Asterisks indicate statistically significant differences compared with 2-B plants: *P<0.05, **P<0.01, ***P<0.001.

Regression analysis (Figs 2 and 4) was performed with SigmaPlot 11 for Windows 7. Where comparisons were made between two treatments (Figs 2–5), data were subjected to one-way ANOVA using SigmaPlot 11 for Windows 7. The variance was analyzed by Tukey’s significant difference, with the level of significance set to *P*<0.05.

## Results

### Two-branched seedlings as a model system for investigating correlative inhibition

These experiments were mostly conducted using 10- to 12-day-old pea seedlings bearing two equal growing cotyledonary shoots (2-B). In 1-B plants one shoot was removed on day 8. Shoot RGR (over a period of 24 h) of *2-B* plants compared with *1-B* plants on day 9 was 0.55 ± 0.03 versus 0.67 ± 0.04 (n=9; *P*<0.05), while the shoot tip weights (shoot apical bud with uppermost leaf, see Fig. 1) on day 12 were 63.4 ± 4.0 mg versus 98.3 ± 4.2 mg (n=10; *P*<0.001). These data show that CI in our system was not strong; the removal of one branch resulted in a 22% increase in RGR in the remaining branch. An explanation for this is that 2-B plants have synchronously growing branches, in contrast to previous experiments on plants with two unequal shoot systems (dominant/inhibited branches) (Morris, 1977; Li and Bangerth, 1999). However, as will be seen, the differences observed in IAA and CK transport and content between 2-B and 1-B plants were much greater, and the explanation for this became the main objective of our study.

### Estimation of IAA export from shoots

IAA export from shoots was determined by a diffusate method, and we specify this export as IEA (Li and Bangerth, 1999). To optimize the sampling of diffusible IAA, we tested the relevance of some factors in the course of diffusible IAA collection.

Shoot diffusates into water at pH 6 (Kotov and Kotova, 2000; Kojima *et al.*, 2002) revealed a 2-fold difference in the amount of diffusible IAA between 1-B and 2-B 10-day-old plants (Fig. 1A). Diffusion from both 1-B and 2-B shoots was stable only throughout the first 2 h of the incubation (Fig. 1B) and decreased with time, coming to a stop after 6 h (Fig. 1A). Diffusion into 60 mM potassium phosphate buffer (pH 6.8) (Johnson and Morris, 1989; Prusinkiewicz *et al.*, 2009; Balla *et al.*, 2011) resulted in a similar decrease in IAA diffusion (Fig. 1B). The use of phosphate buffer resulted in a slightly suppressed IAA diffusion for the first hour, relative to the use of distilled water (Fig. 1B). This effect might be explained by initial neutralization of the apoplastic solution changing the diffusion of IAA according to the chemiosmotic model for auxin transport (Friml, 2010).

To check whether the observed decline in IAA diffusion was not caused by damage to the stem tissue during the long period of contact with aqueous media, we recut the basal end of the shoot tip every 2 hours. This procedure did not affect IAA export (Fig. 1A). The addition of 1% sucrose to the diffusion media transiently promoted IAA diffusion (Fig. 1B). The addition of 10 µM BA, which activates IAA export/synthesis in shoot apices (Li and Bangerth, 1992; 2003), increased the initial IAA diffusion rate from the shoot tips, although all rates declined over time (Fig. 1A). It can be concluded that the decline of IAA export from excised shoots does not appear to be affected by the medium or caused by assimilate or CK deficiency.

We can speculate that the pool of transportable IAA contained in shoot cells, at a constant rate of replenishment, could be quickly exhausted by a greatly enhanced capacity to transport IAA, thus leading to the observed drop in IAA diffusion from the shoot tips. Therefore, the IAA export measured in diffusates may most likely be related to the IAA that was previously accumulated in auxin-transporting cells rather than to the real values of IAA export occurring *in vivo*. Presumably, IAA concentrated in the transport pool can determine the rate of auxin transport, which, in turn, must be equivalent to the rate of refilling of this pool by IAA synthesis; hence, auxin synthesis, the transport pool, and export/transport should be dynamically interrelated.

Compared with the IAA diffusion from shoots of 10-day-old plants, the diffusion from excised 12-day-old shoots containing a mature leaf and shoot tip was stable during the first 3 h (Fig. 1C). The removal of the leaf did not affect the initial IAA diffusion rate but reduced the period of stable IAA diffusion to 2 h (Fig. 1C), as was observed for 10-day-old shoot tips (Fig. 1B, open squares). Mature leaves do not appear to be involved. Therefore, the diffusable IAA collected into distilled water from the shoot tip during the initial 2 h stable period of diffusion can objectively be characterized as active IAA export from the whole shoot. In the current study, this 2 h testing procedure was used as the basis for estimation of shoot IEA.

### Mutual inhibition of IAA export from shoot between two branches of pea

As mentioned above, the removal of one branch in 10- and 12-day-old 2-B plants (to produce 1-B plants) led to approximately double the IAA export from the remaining branch, thereby compensating for the loss of one IAA source (Fig. 2A, B, D). This indicates CI of IEA, and of growth, between the two branches in our pea plant model. In 2-B plants with unequal shoots, the IEA of the dominant shoot was of an intermediate level between that of the shoots of 2-B plants with equal growing shoots and 1-B plants (Fig. 2B, D). The IEAs of shoots from 16-day-old 2-B and 1-B plants were approximately the same (Fig. 2C, D), with values close to those of the shoots of 10- and 12-day-old 2-B plants. These data suggest an age-dependent loss of CI in two-shoot plants.

It appears that in 10- and 12-day-old plants, irrespective of shoot number, the total export of IAA from shoots was equal (Fig. 2A, B, D). To examine whether the shoot IEA value is the result of shoot IAA transported from the shoots, rather than just a response to loss of a shoot, the IAA export from one shoot of 12-day-old 2-B plants was stimulated by BA introduced to the vascular stream. The increase in IEA of the BA-treated shoot was accompanied by a decrease in IEA from the untreated shoot (Fig. 3). Interestingly, short-term incubation of cut shoots in 10 µM BA solution was less effective in increasing IEA (increase of ~25%; see Fig. 1A) than the long-term experiments where the vascular supply of 10 µM BA resulted in a 4-fold increase in IEA (Fig. 3), suggesting that the CK effect needs time to develop.

A 10-fold increase in BA concentration resulted in only a 27% increase in shoot apex weight, even though it induced a 2-fold increase in IAA export (Fig. 3). Similarly, the removal of one branch led to a 2-fold increase in the IEA of the remaining branch (Fig. 2A, B, D), but only slightly activated its growth (see above). It is possible that the shoots in our 2-B model plants were not deficient in CK supply and their growth was close to the maximum limit. Hence, even a small increase in the shoot growth activity would require a substantial increase in CK supply, and possibly the saturation in the growth response occurs before the saturation of the IEA response. Nevertheless, in the experiments with 0 or 1 µM BA treatments, the shoots differing significantly in IEA had similar fresh weights. Such results may be a consequence of the low sensitivity of the method for assaying growth in this case, in comparison with the early estimations of shoot RGR or measurements made 4 days after treatment instead of the 2 days used here. Previous work has shown the activation of shoot IEAs by exogenous CK (Li and Bangerth, 1992; 2003), and so we further examined in detail the involvement of endogenous CKs in the establishment of CI in two-shoot plants.

It is well established that the roots play a major role in regulating shoot branching, and we tested the possibility of root CK being regulated by shoot-derived auxin. Unexpectedly, the levels of diffusible IAA obtained from intact 2-B and branchless (0-B) seedlings previously de-rooted 1.5 cm below the cotyledons were low and similar (Fig. 2D, right, in H_2_O). Thus, IAA transport from the shoots did not follow into the root. The use of 0.5 mM EDTA to estimate IAA translocated along the phloem (Kotov and Kotova, 2000; Jager *et al.*, 2007) gave similar results (Fig. 2D, right, in EDTA). This is consistent with observations that decapitation did not change the IAA content (Kotova *et al.*, 2004) in pea roots, and that the expression of the IAA-regulated genes *PsIPT1* and *PsIPT2* was not detected in the roots after decapitation (Tanaka *et al.*, 2006). It was previously shown in 2-B pea plants that radiolabeled IAA applied on the shoot apex can be transported and accumulated into roots (Morris, 1977; Li and Bangerth, 1999). However, in these studies, root was defined as all plant parts below the cotyledons, which included the hypocotyl as well as the root itself.

### Number of branches in pea model plants determines the xylem CK levels due to activity of the hypocotyl

ELISA of xylem sap from 10-day-old 2-B and 0-B pea plants showed low relative contents of isopentenyladenine (iP)-type CKs, comprising 3–10% of tZ-type CKs, and showing ZR to be a dominant tZ-type CK (Fig. 4D). Our data are in agreement with other literature data for pea plants (Beveridge *et al.*, 1997; Foo *et al.*, 2007; Kotov and Kotova, 2015).

Gradual removal of shoots from 2-B pea plants resulted in pronounced increases in tZ-type CKs in xylem sap obtained from the root plus hypocotyl compared with sap obtained from the root itself (Fig. 4A–С). There was a small but significant effect of shoot-derived auxin on root CK synthesis (Fig. 4C); however, this was much smaller than the effect in the hypocotyl. Therefore, hypocotyl, rather than root, is the major determinant involved in the response to branch reduction through the regulation of xylem CK levels. This conclusion is consistent with the observations showing that shoot IAA is apparently not transported to the root (Fig. 2D, right; Tanaka *et al.*, 2006). The levels of tZ-type CKs in 2-B plants were similar in xylem sap collected from root with or without hypocotyl, and differed only after shoot excision (Fig. 4C). These data suggest that the roots most likely play a role in supplying CK to the upper plant parts, independent of shoot IAA export.

Although our results show that shoot-derived auxin does not have a major role in regulating root CK synthesis, the up-regulation of root CK synthesis by nitrate can play a key role in regulating branching in Arabidopsis and tomato (Takei *et al.*, 2001; Rahayu *et al.*, 2005); however, in rice, nitrogen enhanced the amount of CK by promoting the expression levels of *Os*IPTs in stem nodes rather than in roots (Xu *et al.*, 2015). Root CK may provide a necessary minimum CK supply for the shoot. This is consistent with our data in which a high IAA export from one branch was not able to completely suppress the IEA of another branch (Fig. 3), suggesting that this residual export of IAA might be supported by the root CK. Furthermore, in experiments with strigolactone-deficient pea mutants, apical auxin applications reduced bud growth in de-rooted nodal explants, but had a smaller effect in rooted decapitated plants (Young *et al.*, 2014), in agreement with a growth-promoting role for root CK which may be largely independent of shoot auxin.

Our results indicated that the levels of the hypocotyl-derived tZ-type CKs in ascending xylem sap entering shoots were four times higher in 1-B than in 2-B plants (Fig. 4A, C). Together with the stimulation of shoot IEA by exogenous CK (Fig. 3), the data provide strong evidence that xylem CK controls shoot IEA and possibly shoot growth as well. The results also show that the number of branches in our model plants determined xylem CK levels, and that this effect is controlled by the CK-synthetic activity of the hypocotyl.

### A dynamic model for auxin–cytokinin inter-regulation in two-branched pea plants

In order to account for how shoots can regulate the levels of xylem CK in our model plants, we analyzed the contents of IAA and CKs in the hypocotyl. As expected, in 0-B plants the IAA level in the hypocotyl was rather low (Fig. 5), indicating that previously accumulated auxin could not be transported to the roots (Fig. 2D) and was most likely degraded. The dramatically increased concentrations of tZ-type and iP-type CKs in the hypocotyls of 0-B plants (Fig. 5) were consistent with published accounts of the role of auxin in the negative regulation of CK levels (Li *et al.*, 1995; Kotova *et al.*, 2004; Takei *et al.*, 2004; Nordstrom *et al.*, 2004; Tanaka *et al.*, 2006; Dun *et al.*, 2012; Kotov and Kotova, 2015). In 0-B plants, the levels of tZ-type CKs were highly increased compared with 2-B plants both in xylem sap obtained from root plus hypocotyl (Fig. 4A, C) and in hypocotyl tissue (Fig. 5). However, this increase was 3-fold less for xylem sap from roots (Fig. 4A, C), thus implying the hypocotyl origin of xylem CK.

**Fig. 6.**
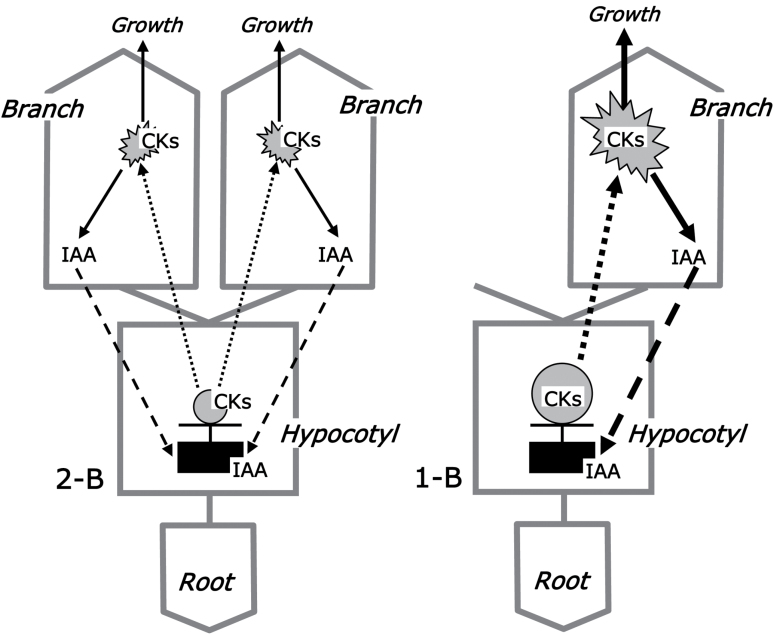
Schematic representation of the IAA–CK balance in the model system of pea seedlings. Roots were shown not to be involved in the hormonal interactions (Fig. 2D, right side; Fig. 4A–C). The interaction between IAA and CKs occurs by transport of these hormones. Xylem CK (dotted lines) acts positively on the production/export of shoot auxin (dashed lines) (Fig. 3; Li and Bangerth, 1992, 2003), while IAA acts negatively on the synthesis/content of CKs in hypocotyl (0-B variant in Fig. 5; Li *et al.*, 1995; Tanaka *et al.*, 2006; Kotov and Kotova, 2015). Our data suppose that IAA homeostasis in the hypocotyl is key to the formation of the IAA–CK balance; a steady-state tissue IAA level is sustained near a threshold level, below which CK synthesis is switched on, activating the export of IAA from shoot(s) through ascending xylem flow of CKs. The removal of one shoot from 2-B plants results in an initial decrease of hypocotyl IAA content that triggers CK synthesis, which continues until the concentration of CKs in hypocotyl (Fig. 5) and, as a consequence, in xylem sap (Fig. 4A, C) becomes sufficient to increase IAA export from the remaining shoot (Fig. 2A, B, D). This compensates for the loss of a shoot, restoring the IAA concentration in the hypocotyl, and stopping further CK synthesis and elevation of xylem CK levels.

In hypocotyls of 1-B and 2-B plants, IAA levels were equal (Fig. 5). This can be explained if the total IEA from two shoots in 2-B plants equals that from one shoot in 1-B plants (Fig. 2A, D). At the same time, the concentrations of tZ-type CKs were dissimilar in 1-B and 2-B plants (Fig. 5), similar to the findings observed in xylem sap (Fig. 4A, C). This observation is difficult to reconcile with the potential for IAA to regulate CK levels, as shown above for 0-B plants. To explain this paradox we propose a dynamic interaction model that includes the transport of IAA and CK along with the IAA–CK interactions (Fig. 6).

We assume that the IAA homeostasis observed in the hypocotyl is being maintained by shoot IAA, which is positively controlled by xylem CK synthesized mainly in the hypocotyl, under negative auxin control. In this model, the hypocotyl IAA content can be sustained at a constant level, below which auxin-regulated CK synthesis will be switched on. The resulting increase in xylem CK levels returns IAA content to the previous level through CK-activated IAA export from shoots. Accordingly, the removal of one shoot from a 2-B plant will decrease IAA content in the hypocotyl and so triggers CK synthesis, which continues until CK levels in xylem sap are enough to induce IAA export from the remaining shoot. This export restores the IAA level in the hypocotyl and stops further CK synthesis (Fig. 6).

## Discussion

### Correlative inhibition in the two-branched pea model system is age dependent

When studying 2-B plants at various ages, we found that differences in shoot IEA between 2-B and 1-B plants, and hence CI in this system, disappeared in 16-day-old plants bearing two mature leaves (Fig. 2A–D). Similar loss of CI has also been observed in two-shoot plants bearing equal shoots with three mature leaves (Li and Bangerth, 1999. Thus, it is evident that shoots develop independence. Similarly, as the branches grow, they eventually became resistant to strigolactones, which inhibit the outgrowth of axillary buds (Dun *et al.*, 2013). However, in 2-B pea plants with unequal shoots, removal of the dominant shoot results in growth and IEA activation in the subordinate shoot (Li and Bangerth, 1999) which suggests that the ability of branches to compete does not disappear completely with increasing age. It appears that if a shoot/branch lags behind others and thus remains physiologically young it can remain responsive to the CI stimuli.

### Mutual inhibition between two branches of pea occurs according to the second messenger model

Our studies, as in the experiments of Li and Bangerth (1999), lead us to similar basic conclusions, namely, that competition between branches on the same plant occurs at the level of their IEA. A clear understanding of this variable and the reasons leading to its regulation is the key to elucidating the CI phenomenon. IEA reflects IAA synthesis, yet is also dependent on quantitative regulation of IAA transport. The latter follows from the results showing that IAA contents in the hypocotyl (Fig. 5) were directly correlated with the shoot IEA (Fig. 2A, D). The correlation obtained between IEA (Fig. 2A, D) and xylem CK (Fig. 4C) in 1-B and 2-B plants on the one hand, and the ability of ВА supplied to the vascular stream to affect shoot IEA (Fig. 3) on the other hand, together argue an important role for endogenous CK signaling in the shoot/branch competitive interaction, at least in our model 2-B system. This possibility is supported by the observations of Li and Bangerth (2003) and of CK-driven polarization of *Ps*PIN1 proteins in pea axillary buds (Kalousek *et al.*, 2010). Localizing the hypocotyl as the site of control of acropetally transported CKs (Fig. 4), which in turn regulate the shoot IEA (Fig. 3), has suggested a model in which CI is a function of an IAA–CK feedback loop, where CK essentially acts as a second messenger for IAA (Fig. 6).

The phenomenon of CI has led to alternative auxin transport models (Li and Bangerth, 1999; Bangerth *et al.*, 2000). Li and Bangerth (1999) proposed that IAA transported from a dominant shoot can somehow competitively impede IAA outflow from subordinate shoots [auxin-transport autoinhibition (ATA) at junctions]. Li and Bangerth (1999) did not find significant differences in auxin transport capacity between subordinate and dominant shoots, reporting only 20% elevation in internodes of the dominant shoot. Therefore, they considered ATA only as a result of a possible competition at the level of auxin transport and not a change in auxin transport capacity. However, we showed a substantial increase in the xylem CK level after shoot removal in 2-B pea plants, and that could enhance auxin transport from a residual shoot.

Although it is possible to explain ATA using a second messenger model, other direct action models have been proposed. The auxin transport experiments with Y-shaped pea explants demonstrated the autoinhibition of ^3^H-IAA transport in one arm by the simultaneous transport at various unlabeled IAA concentrations in the other arm, but high (unphysiological) concentrations of IAA (up to 50 mM IAA in lanolin) were needed to achieve reasonable autoinhibition (Li and Bangerth, 1999). Next, in the more sensitive ‘endogenous ATA’ system, it was shown that the transport of ^3^H-IAA through pedicels of young tomato fruits decreased with increases in the concentration of unlabeled IAA from 1 to 50 µM in the agar receiver (Bangerth *et al.*, 2000). In our study, the IAA contents in the hypocotyl were similar in 1-B and 2-B plants (Fig. 5), whereas the shoot IEAs were different (Fig. 2A, D), a fact that is difficult to reconcile with the ATA effect. Further, our data showed that high IAA export from one branch does not completely inhibit export from another branch (Fig. 3); this observation does not agree with the model outlined by Prusinkiewicz *et al.* (2009), which predicts that high auxin flux from one source should fully shut down auxin flux from another source. We believe that our data from 2-B pea are consistent with the second messenger model for auxin action, rather than the auxin transport model. However, data from other systems, discussed below, could be consistent with the auxin transport model.

### Auxin-induced bud inhibition mechanisms in other model systems

Müller *et al.* (2015) reported that in Arabidopsis plants CK is not necessarily required to promote bud outgrowth. This conclusion followed from experiments indicating that the axillary buds in nodal explants of CK mutants defective in CK synthesis (*ipt 3*, *5*, *7*) and signaling (*arr3*, *4*, *5*, *6*, *7*, *15*), grew equivalent to wild-type buds. The authors suggested that CKs play little part in auxin-mediated bud repression, which is consistent with the canalization-based hypothesis for bud activation (Prusinkiewicz *et al.*, 2009). In Arabidopsis, the activity of СKs to up-regulate polar transport of IAA has not yet been reported. Conversely, in Arabidopsis roots CK was found to regulate endocytic recycling of the auxin efflux carrier PIN1 by redirecting it for lytic degradation in vacuoles (Marhavý *et al.*, 2011). This suggests a negative role of CK in auxin transport regulation, but it has yet to be determined whether this phenomenon occurs in overground parts of Arabidopsis plants.

Auxin-induced bud inhibition observed in decapitated plants or nodal explants is different from bud dormancy in intact plants. In decapitated systems, axillary bud growth usually cannot be stopped completely even under high concentrations of applied apical auxin. It appears that auxin treatment suppresses only the beginning of bud growth, which, once started, continues even if slightly restrained by apical auxin (Chatfield *et al.*, 2000; Kotov and Kotova, 2010). In addition, the inhibitory effect of apical auxin was found to be largely dependent on the bud size, decreasing with an increase in size (Chatfield *et al.*, 2000; Kotov and Kotova, 2010), which has periodically raised the question of whether auxin is the hormone of apical dominance (Cline and Sadeski, 2002; Mason *et al.*, 2014). Moreover, the bud inhibition by exogenous auxin in decapitated pea plants was often associated with bud swelling that did not occur in the dormant buds in intact wild-type plants (Beveridge *et al.*, 2000). The possible reason for these observed anomalies could be connected to increased cellular osmotic pressure, which has proved to be up to 0.15 MPa higher in the buds of decapitated plants treated with auxin than that of intact plants (Kotov and Kotova, 2010), with the difference obtained being equivalent to 50 mM (1.8 %) sucrose solution. These data suggest that the suppression of bud growth by exogenous auxin most likely occurs against a background of an increased amount of assimilates/sugars, the transport of which to the buds was shown to be activated following the removal of a dominant sink organ, and which has the potential to promote bud outgrowth (Mason *et al.*, 2014; Barbier *et al.*, 2015). Gene expression profiling in Arabidopsis suggests that the sugar-repressive element (SRE) is one of the potential regulatory elements involved in the down-regulation of gene expression after decapitation, and SRE may contribute to the nutritional regulation of gene expression in Arabidopsis axillary buds (Tatematsu *et al.*, 2005). Most likely, the independence of axillary bud growth in nodal Arabidopsis explants from СKs (Müller *et al.*, 2015) could be accounted for by the availability of sugars (Barbier *et al.*, 2015).

## Abbreviations:

ATA: auxin transport autoinhibition
0-B: branchless plants
1-B: one-branched plants
2-B: two-branched plants
BA: 6-benzylaminopurine
CI: correlative inhibition
CK: cytokinin
IAA: indole-3-acetic acid
IEA: IAA export activity
iPR: isopentenyladenosine
iP-type CK: isopentenyladenine-type cytokinin
PBS: phosphate-buffered saline
PVPP: polyvinylpolypyrrolidone
RGR: relative growth rate
tZ: *trans*-zeatin
ZR: zeatin riboside.
